# Case Report: Anti-PD-1 therapy as a catalyst for the rapid transformation of a hepatic nodule into HCC: a “soil and seed” paradox in metachronous triple primary malignancies?

**DOI:** 10.3389/fimmu.2026.1831238

**Published:** 2026-05-01

**Authors:** Xian Yang, Dehua Kong, Boan Lai, Chuan Zhang, Na Li

**Affiliations:** 1 Department of Oncology, Suining Central Hospital, Suining, Sichuan, China; 2 Department of Pathology, Suining Central Hospital, Suining, Sichuan, China

**Keywords:** cancer immunoediting, hepatocellular carcinoma, immune checkpoint inhibitors, multiple primary malignancies, soil-catalyst model

## Abstract

Multiple primary malignancies (MPMs) are increasingly recognized, yet the impact of immune checkpoint inhibitors (ICIs) on the evolution of metachronous tumors remains poorly understood. Here, we report a rare case of metachronous triple primary malignancies—lung squamous cell carcinoma, gastric adenocarcinoma, and hepatocellular carcinoma (HCC)—in a 68-year-old male with chronic HBV infection and a history of heavy tobacco and alcohol use. Notably, while receiving the PD-1 inhibitor sintilimab for gastric adenocarcinoma, a previously stable hepatic nodule underwent rapid malignant transformation into histologically confirmed HCC. This paradoxical progression occurred despite the patient achieving a partial response in the gastric lesion and sustained remission of the lung malignancy. Given the patient’s high-risk hepatic background, this case raises the hypothesis that ICIs may inadvertently disrupt local immune tolerance in a predisposed “pro-oncogenic soil, ” potentially catalyzing the transition of dormant pre-neoplastic lesions. This report underscores the need for rigorous baseline liver assessment and close radiological surveillance in high-risk patients undergoing immunotherapy, and highlights the importance of further research into the organ-specific effects of ICIs.

## 1 Introduction

Multiple primary malignancies (MPMs) are defined as the occurrence of two or more histologically distinct primary malignant tumors in a single individual, arising either synchronously or metachronously, independent of metastatic spread (1). Driven by advancements in diagnostic imaging and prolonged cancer survival, the incidence of MPMs has risen steadily, now reported in 0.7% to 18.4% of cancer patients (1–3). The pathogenesis of MPMs is multifactorial, involving genetic predisposition (4, 5), shared environmental exposures (6), and iatrogenic factors such as prior radiotherapy (7, 8) or chemotherapy (9). Beyond their clinical complexity, MPMs provide a unique biological model for investigating shared tumorigenic mechanisms and the challenges of long-term oncological management.

Immune checkpoint inhibitors (ICIs) have revolutionized the treatment landscape for diverse solid tumors, including lung, gastric, and hepatocellular carcinomas (HCC), significantly extending overall survival (10). However, the widespread use of ICIs has also expanded the spectrum of immune-related adverse events (irAEs) (11, 12). While established irAEs—such as pneumonitis, colitis, and hepatitis—are typically characterized as inflammatory damage to normal tissues due to a loss of immune tolerance (13, 14), the potential for ICIs to alter the evolutionary trajectory of pre-existing lesions (15) or promote *de novo* malignancies (16, 17) remains largely underexplored.

Current literature regarding the impact of ICIs on secondary tumorigenesis remains equivocal. Some retrospective data suggest that enhanced immune surveillance may reduce the risk of second primary tumors (18). Conversely, emerging case reports describe the development of histologically distinct malignancies even while the index tumor is under robust therapeutic control (16). This paradoxical phenomenon challenges the conventional paradigm that immunotherapy exclusively promotes beneficial anti-tumor responses. It suggests that ICIs might inadvertently disrupt systemic immune homeostasis, potentially creating a localized microenvironment conducive to the accelerated growth of a secondary malignancy.

**Figure 1:**
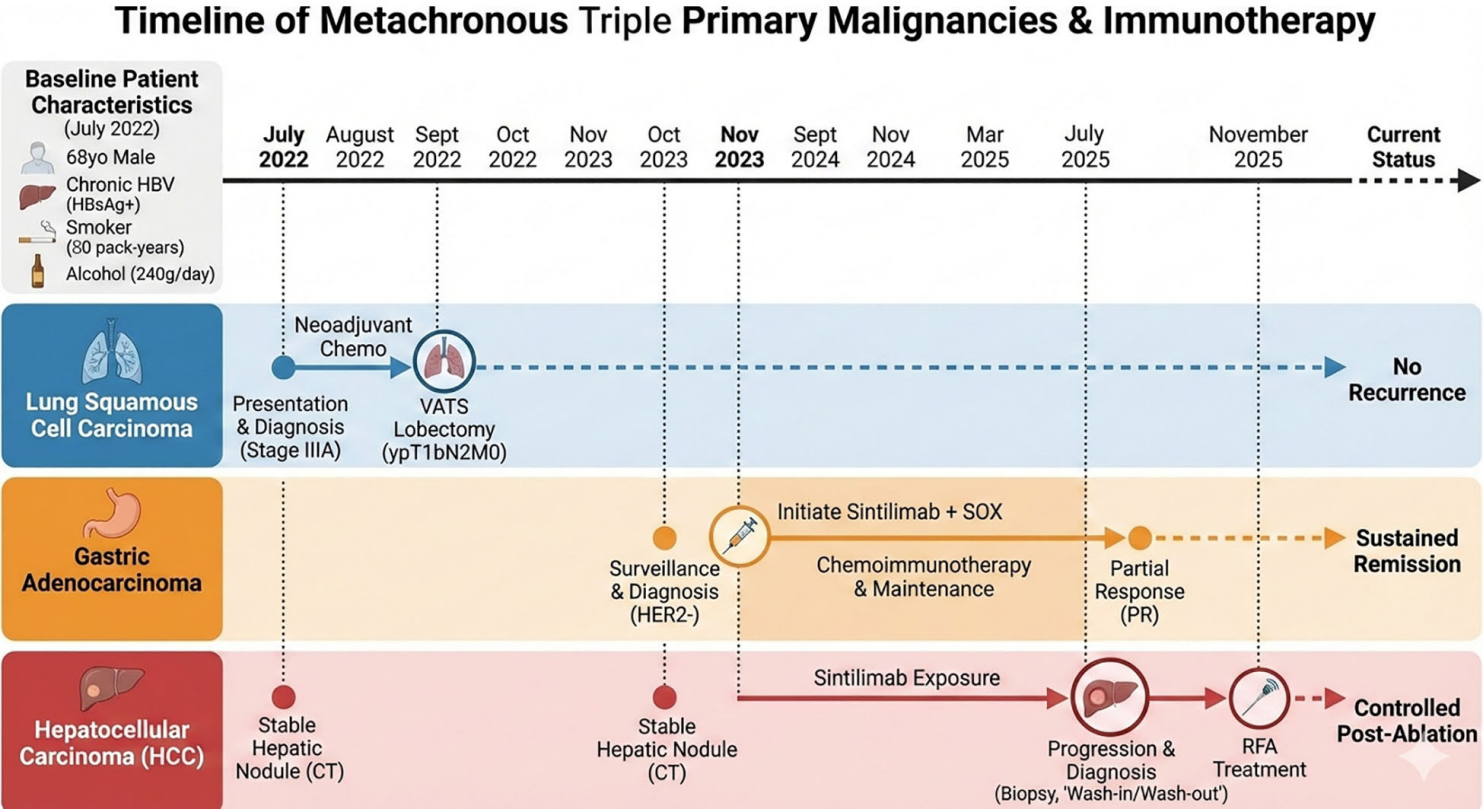
Clinical timeline of metachronous triple primary malignancies and sequential therapeutic interventions. The timeline summarizes the sequential diagnosis, systemic therapy, surgical and local intervention strategies for a patient with triple primary malignancies.

In this report, we present a complex case of metachronous triple primary malignancies—lung squamous cell carcinoma, gastric adenocarcinoma, and HCC—in which the HCC emerged *de novo* during PD-1 inhibitor therapy combined with chemotherapy. Notably, a previously stable hepatic nodule underwent rapid malignant transformation into HCC, while the patient’s lung cancer remained in surgical remission and the gastric adenocarcinoma demonstrated a sustained response (**Figure 1**). This distinct temporal association and organ-specific divergence suggest a potential “soil and seed” paradox, where ICI therapy may have accelerated a dormant neoplastic process in the liver. By integrating longitudinal clinical and radiological evidence, we discuss the possible immunobiological mechanisms underlying this under-recognized association to provide a cautionary framework for clinical practice.

## 2 Case presentation

### 2.1 Case description

A 68-year-old male presented in July 2022 with a six-month history of progressive cough and expectoration. The patient’s medical history was notable for chronic hepatitis B virus (HBV) infection (HBsAg-positive carrier, >250 IU/ml; HBV DNA: 238.8 IU/ml). His social history included significant tobacco (80 pack-years) and alcohol consumption (240g/day for 30 years), both discontinued one year prior to admission. He had a surgical history of traumatic liver rupture over 30 years ago. Family history was negative for malignancies.

### 2.2 Initial malignancy: lung squamous cell carcinoma

**Figure 2:**
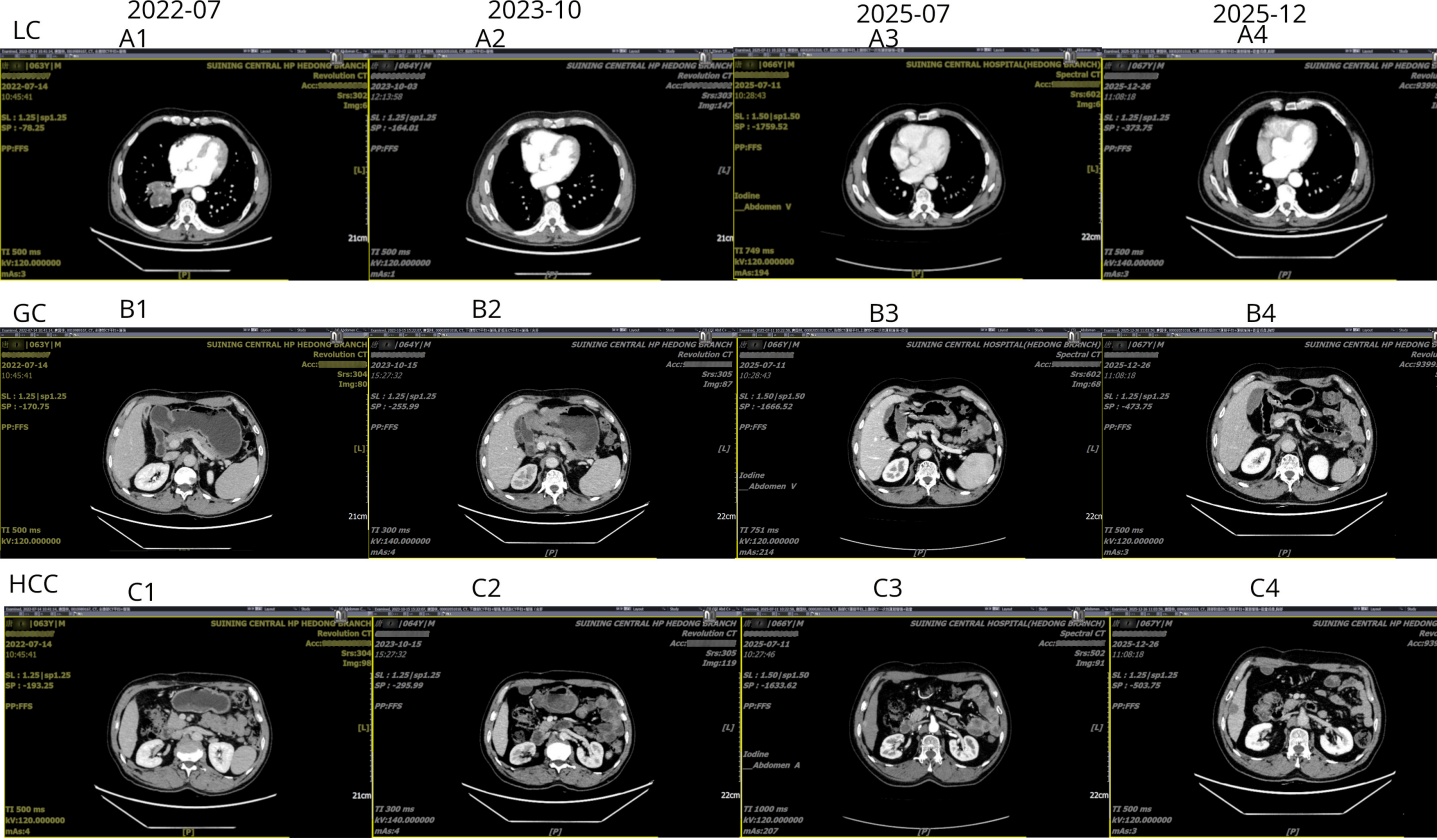
Serial CT imaging of metachronous triple primary malignancies over the clinical course. Serial CT scans illustrate the clinical course of lung cancer (row A, diagnosed July 2022), gastric cancer (row B, diagnosed October 2023), and hepatocellular carcinoma (row C, diagnosed July 2025), with latest follow-up in December 2025.

**Figure 3:**
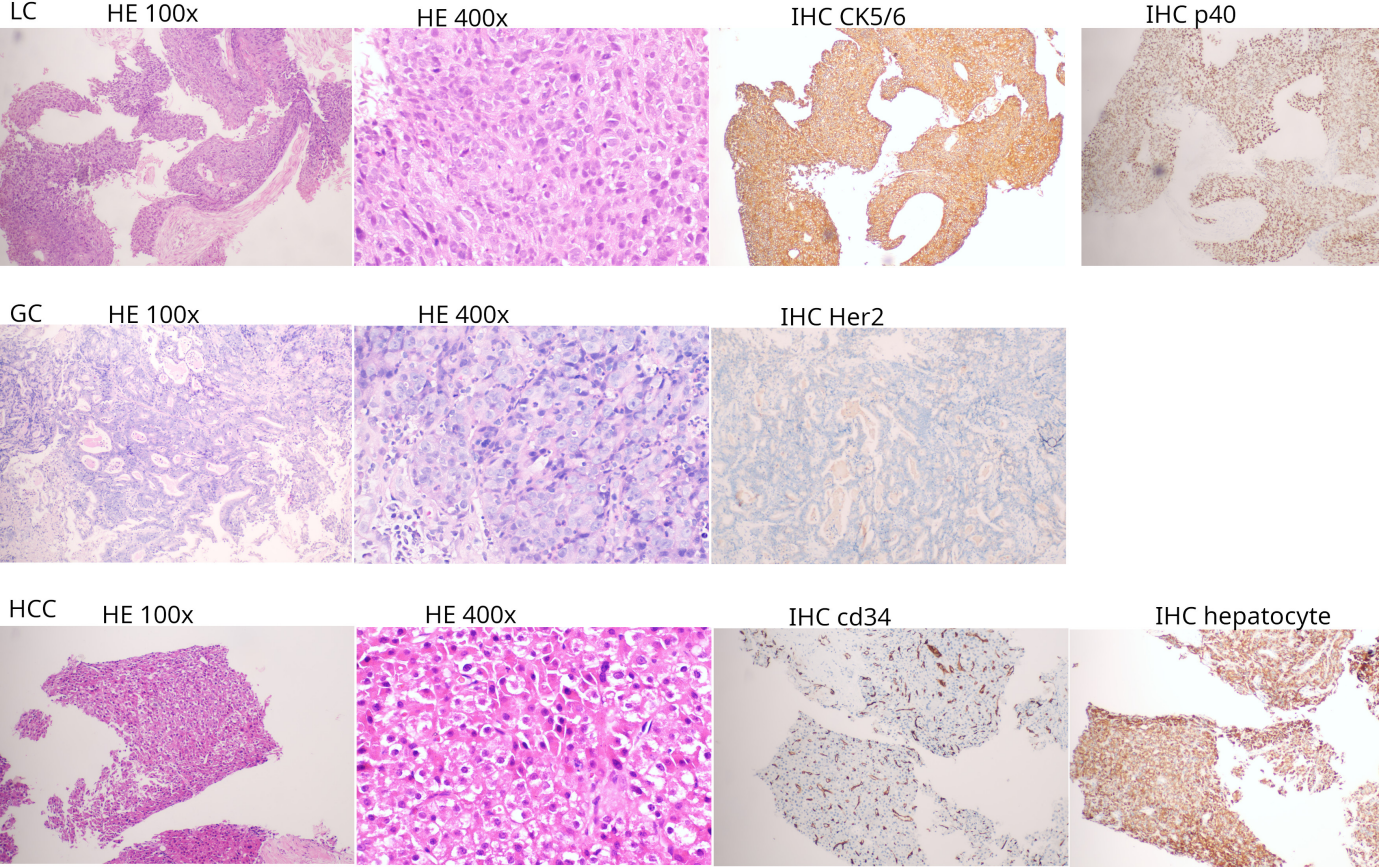
Histopathological and immunohistochemical features of the three primary tumors. HE and IHC staining profiles for each malignancy. LC (row A): squamous cell carcinoma confirmed by CK5/6 and p40 positivity. GC (row B): gastric adenocarcinoma with negative HER2 expression. HCC (row C): hepatocellular carcinoma confirmed by CD34 and hepatocyte marker expression.

In July 2022, Chest CT imaging revealed a mass in the right lower lobe (**Figure 2** A1). Bronchoscopic biopsy and immunohistochemical staining (CK5/6+, P40+) confirmed lung squamous cell carcinoma (**Figure 3**). A baseline contrast-enhanced abdominal CT identified a stable, faintly enhancing nodule in the superior segment of the right posterior hepatic lobe (**Figure 2** C1). Following two cycles of neoadjuvant paclitaxel/carboplatin, the patient underwent video-assisted thoracoscopic surgery (VATS) right middle and lower lobectomy. Pathological staging was ypT1bN2M0; no adjuvant therapy was administered.

### 2.3 Second malignancy: gastric adenocarcinoma

In October 2023, routine follow-up CT detected a gastric antrum lesion (**Figure 2** B2). Gastroscopic biopsy confirmed HER2-negative gastric adenocarcinoma. The following month, the patient initiated first-line chemoimmunotherapy with sintilimab plus SOX every 3 weeks. A baseline abdominal CT showed the previously identified hepatic nodule was stable (**Figure 2** C2). After six cycles, the patient achieved a partial response (PR) and transitioned to sintilimab monotherapy.

### 2.4 Third malignancy: hepatocellular carcinoma

In July 2025, during sintilimab maintenance, the patient presented with anorexia and fatigue. Contrast-enhanced CT revealed the hepatic nodule had progressed to 1.0 cm, showing classic wash-in and wash-out enhancement consistent with HCC (**Figure 2** C3). While serum alpha-fetoprotein remained negative, ultrasound-guided biopsy with immunohistochemistry confirmed HCC, showing Hepatocyte+, GPC3+, HSP70+ and CD34+ sinusoidal capillarization (**Figure 3**). The patient underwent successful radiofrequency ablation on November 5, 2025.

### 2.5 Outcome and follow-up

Currently, all three primary malignancies remain stable: the lung carcinoma shows no recurrence, the gastric adenocarcinoma is in sustained remission, and the HCC is controlled post-ablation. The patient maintains an ECOG performance status of 1.All events were listed in the timeline (**Figure 1**).

## 3 Discussion

This case reports a rare metachronous triple primary malignancy in a patient with chronic hepatitis B virus (HBV) infection and a long history of heavy tobacco and alcohol use, who was successively diagnosed with lung squamous cell carcinoma, gastric adenocarcinoma, and subsequently developed hepatocellular carcinoma (HCC). The most clinically distinctive feature of this case is as follows: a hepatic nodule that had remained radiographically stable for a long period prior to immunotherapy, underwent rapid malignant transformation during maintenance therapy with the PD-1 inhibitor sintilimab, and was histopathologically confirmed as HCC. Meanwhile, the patient’s previously diagnosed lung and gastric tumors remained well-controlled.

It is well established that chronic HBV infection and long-term excessive alcohol consumption are independent risk factors for HCC, and they also have a synergistic pathogenic effect (19–21). Individuals with dual exposure have a significantly elevated baseline risk of developing liver malignancy. In routine clinical practice, newly developed HCC in such high-risk individuals is often attributable to the natural disease progression of the underlying liver disease, which is a plausible explanation for the malignant transformation observed in this patient.

Nevertheless, the striking temporal association—a previously stable hepatic nodule undergoing rapid malignant transformation during treatment with the PD-1 inhibitor sintilimab—prompts the hypothesis that immune checkpoint inhibitors (ICIs) may, under specific conditions, accelerate *de novo* hepatocarcinogenesis. Although ICIs are intended to restore anti-tumor immunity, the phenomenon of “hyperprogressive disease” (HPD) indicates that PD-1/PD-L1 blockade can paradoxically accelerate tumor kinetics (22, 23). While HPD is typically described in the context of established tumors, the underlying biological principles—disruption of immune equilibrium and unintended stimulation of oncogenic pathways—may also apply to the promotion of nascent malignancies (24). We postulate that the malignant transformation observed herein may have resulted from the interplay between the patient’s predisposing hepatic microenvironment and the immunological perturbations induced by ICI therapy. Several potential mechanisms may explain this acceleration, operating at different but interconnected levels.

### 3.1 Several potential mechanisms

#### 3.1.1 Exacerbation of chronic inflammation in the hepatic microenvironment

Cancer-related inflammation is central to hepatocarcinogenesis. The liver is an immunologically unique, tolerogenic organ, yet highly susceptible to chronic, low-grade inflammation, a recognized prerequisite for malignant transformation (25). The patient’s chronic HBV infection and prolonged alcohol consumption had likely established a microenvironment characterized by persistent DNA damage and pro-inflammatory cytokine release. ICI administration can induce an inflammatory surge within such a sensitized environment. By disinhibiting the systemic immune system, ICIs may exacerbate pre-existing localized inflammation. Crucially, this exacerbation is likely liver-localized, arising from the organ’s unique tolerogenic properties and pre-existing chronic injury, rather than representing a systemic inflammatory response.This surge can elevate levels of cytokines such as IL-6 and TNF-alpha, which are known to trigger the activation of hepatic stellate cells and oncogenic signaling pathways. In this context, immunotherapy may have acted as a precipitating factor superimposed on chronic hepatitis, accelerating the transition from chronic inflammation to overt malignancy. This inflamed microenvironment thus established a permissive “soil” that primed the liver for subsequent immune-mediated tumor evolution.

#### 3.1.2 Immunoediting: transition from equilibrium to escape

**Figure 4:**
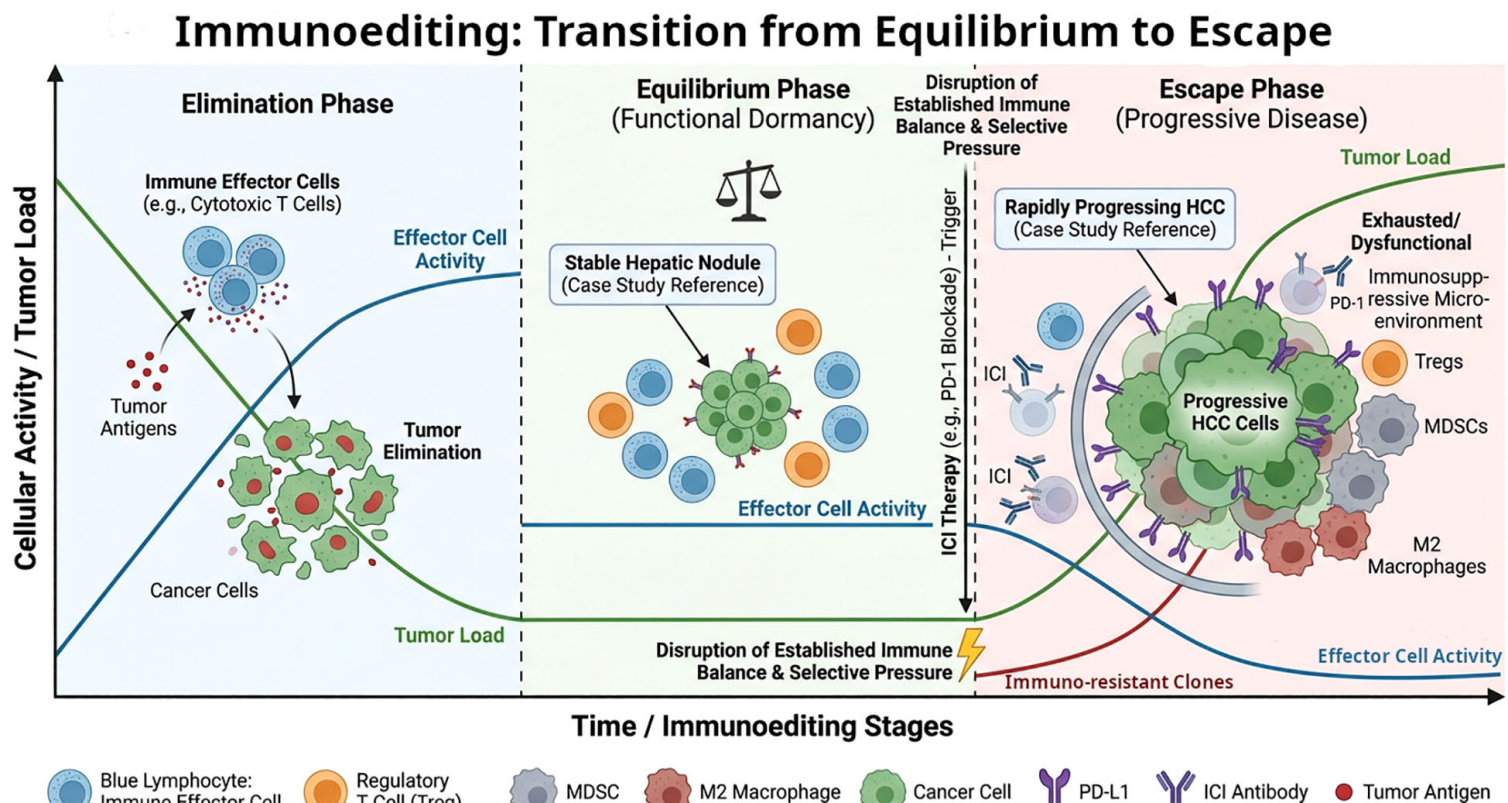
Mechanistic model of ICI-induced accelerated hepatocarcinogenesis. This schematic illustrates the dynamic transition of the hepatic nodule from immune-mediated stability to rapid malignancy. During the Elimination and Equilibrium phases, endogenous immune surveillance maintains the lesion in a state of functional dormancy, corresponding to its long-term radiographic stability. The initiation of ICI therapy (represented by the lightning bolt) acts as a decisive selective pressure that disrupts this established immunological balance. While the therapy successfully targets sensitive clones in other organs, it concurrently facilitates the rapid proliferation of immuno-resistant clones (indicated by the red line) within the unique hepatic microenvironment. This selective advantage allows these resistant populations to bypass local surveillance and enter the Escape Phase, characterized by an exhausted microenvironment and a sharp surge in total tumor load (indicated by the green line), ultimately manifesting as the rapid transformation into HCC observed in this case.

On this background of chronic inflammation, the “cancer immunoediting” hypothesis provides a framework to interpret the dynamic interplay between the hepatic nodule and the immune system. This theory posits that the immune system actively shapes tumor evolution through three phases: elimination, equilibrium, and escape (26). In the equilibrium phase, the immune system successfully prevents a nascent tumor from expanding, maintaining it in a state of functional dormancy or radiographic stability. In this patient, the baseline hepatic nodule likely represented a cell population in the equilibrium phase—constrained, but not eradicated, by endogenous immune surveillance. While sintilimab effectively mediated the elimination of the lung and gastric tumors, it may have exerted selective pressure within the liver. By disrupting the established immune balance, the therapy potentially facilitated the “escape” of occult, resistant hepatocyte clones, allowing a dormant lesion to bypass surveillance and manifest as rapidly progressing HCC (**Figure 4**). This hypothesis directly addresses the core paradox of the case: why a previously stable nodule transformed rapidly during ICI therapy while other tumors responded favorably.

#### 3.1.3 Modulation of the immune balance: effector vs. suppressive populations

Beyond the immunoediting dynamics described above, the specific means by which immune escape is achieved may involve shifts in local immune regulatory balance. ICI efficacy relies on the balance between effector T cells and suppressive populations, such as regulatory T cells and myeloid-derived suppressor cells. Paradoxically, recent evidence suggests that PD-1 blockade can sometimes induce the expansion or hyperactivation of these suppressive subsets (27). In the liver—an organ naturally skewed toward tolerance to prevent overreaction to blood-borne antigens—this remodeling can have unintended consequences. If ICI therapy shifted the local hepatic microenvironment toward a more suppressive state, it may have created an “immune-privileged” niche. This would allow the emerging HCC to proliferate, evading surveillance even while other malignancies responded to treatment. Thus, this mechanism offers a plausible explanation for the organ-specific heterogeneity observed in this case: the same ICI therapy that unleashes effector T cells against lung and gastric tumors may, within the unique hepatic microenvironment, paradoxically expand suppressive populations and facilitate local immune escape. This underscores a critical observation: a therapeutic response in one organ system does not guarantee a uniform anti-tumor effect across the systemic immune landscape.

### 3.2 Limitations

We acknowledge several important limitations. First, genetic characterization of the patient—including germline susceptibility testing and tumor-specific molecular features (e.g., exome sequencing of the hepatic nodule before and after ICI treatment)—was not performed. Such data would have been critical to distinguish between natural disease progression and ICI-accelerated hepatocarcinogenesis. Second, without longitudinal immune profiling of the hepatic microenvironment, the proposed mechanisms (chronic inflammation exacerbation, immunoediting, and shifts in regulatory T cell balance) remain speculative. Therefore, our discussion is intended to generate hypotheses for future studies rather than to establish causation. Third, as a single case report, our findings may not be generalizable. Future larger-scale studies with serial biopsies and molecular analyses are warranted to explore the potential paradoxical role of ICIs in *de novo* hepatocarcino-genesis in high-risk patients.

## 4 Conclusion

In summary, this case highlights a rare trajectory of metachronous triple primary malignancies, characterized by the rapid emergence of HCC during PD-1 inhibitor therapy. The tight temporal correlation suggests that ICIs may act as an evolutionary catalyst in the setting of chronic liver disease, potentially remodeling the local immune landscape to favor hepatocarcinogenesis. Crucially, this case challenges the reliance on diagnostic monism. While metastasis remains the most common etiology for new lesions, the possibility of a second primary malignancy should not be overlooked, particularly in patients with underlying high-risk factors (e.g., chronic hepatitis or cirrhosis) or atypical radiological progression. Moreover, organ-specific tumor markers (e.g., AFP) negativity does not preclude the possibility of a second primary malignancy. Consequently, standard protocols should evolve to prioritize histological confirmation alongside high-resolution imaging. Ultimately, while ICIs remain a cornerstone of oncology, large-scale studies are urgently required to elucidate the mechanisms of organ-specific paradoxical progression and ensure that therapeutic efficacy is balanced by robust long-term safety.

## Data Availability

The original contributions presented in the study are included in the article/supplementary material. Further inquiries can be directed to the corresponding author.
